# Pleurodesis with povidone iodine in patients with malignant pleural effusion in a tertiary center in Nigeria

**DOI:** 10.11604/pamj.2021.38.169.22405

**Published:** 2021-02-15

**Authors:** Benjamin Irene Omoregbee, Stanley Okugbo

**Affiliations:** 1 Department of Cardiothoracic Surgery, Castle Hill Hospital, Cottingham, United Kingdom,; 2 Cardiothoracic Surgery Unit, Department of Surgery, University of Benin Teaching Hospital, Benin City, Nigeria

**Keywords:** Pleurodesis, povidone iodine, malignant, pleural effusion

## Abstract

**Introduction:**

malignant pleural effusion occurs as a consequence of a primary or metastatic malignant process involving the pleura. The aim of pleurodesis is to prevent re-accumulation of the effusion and avoid the need for repeated hospitalization. Povidone iodine has been used in other climes for pleurodesis with good results. The aim of this study is to assess the efficacy and safety of povidone iodine in producing pleurodesis as compared to tetracycline.

**Methods:**

the study is a prospective experimental study. The patients are randomized into two groups A (tetracycline-control) and B (povidone iodine). All patients are assessed with chest X-ray after 1 week and 1 month. The responses were ascribed as complete, partial or failure.

**Results:**

thirty patients were recruited into this study, 15 patients in each group A (tetracycline) and B (povidone iodine). The mean age was 45.7±14.24 years. The commonest primary malignancy was Breast cancer (70%) followed by bronchogenic cancer (10%). Seventy three (73%) of the patients in this study had complete response and in 7% pleurodesis failed whilst 20% has partial response. In the povidone group the success rate was 93.4% and in the tetracycline group was 93.3% with a p-value of 0.716. There was no statistical difference in the responses based on the agents used.

**Conclusion:**

malignant pleural effusion is a devastating condition as it heralds the end-of-life processes of a primary malignancy. Povidone iodine is a safe, cheap, effective, widely available and effective pleurodesing agent for use in patients with malignant pleural effusion.

## Introduction

Pleurodesis is a procedure that creates obliteration of the pleural space by chemical or mechanical means [1,2]. It is frequently considered in the management of malignant pleural effusions and recurrent pneumothoraces. Malignant pleural effusion occurs as a consequence of a primary malignancy of the pleura or secondary involvement of the pleura from a metastatic malignant process [3,4]. Malignant pleural effusion is identified by the presence of malignant cells in the pleural fluid and/or parietal pleura [4]. Malignant pleural effusion accounts for 22% of all pleural effusions, and affects 300,000 patients annually (UK and USA) [5]. Approximately 50% of patients with breast cancer, 25% of those with lung cancer and 90% of those with mesothelioma would develop a symptomatic pleural effusion [5]. In Nigeria, breast cancer is the commonest cause of malignant pleural effusion. A study by Ogunleye *et al*. in Lagos showed that breast cancer was responsible for 47% of cases of malignant pleural effusion followed by bronchogenic cancer (9.4%) and ovarian cancer (4.7%) [6]. However, lung cancer is still the commonest cancer in the world with about 1.35 million new cases worldwide in 2002 and the leading cause of cancer related deaths (1.37 million deaths in 2008) [7]. The aim of pleurodesis in patients with malignant pleural effusion is to prevent re-accumulation of the effusion thereby improving symptoms, quality of life and avoiding the need for repeated hospitalization [1,2]. This can be achieved by both mechanical and chemical means.

Chemical pleurodesis may be the best available treatment for recurrent and troublesome pleural effusions when the underlying cause cannot be corrected. A wide spectrum of pleurodesing agents have been used, but the search for the ideal agent for pleurodesis continues [8]. The ideal agent for pleurodesis must be highly efficacious, should have a high molecular weight, as well as low regional clearance, easily accessible, cheap, easy to administer and well tolerated with minimal or no side effects [9]. Talc, bleomycin and tetracycline are the most frequently used pleurodesing agents in clinical practice worldwide. However, tetracycline is the commonest agent used in most centres in Nigeria. Sterile talc is considered the most effective chemical agent for malignant pleural effusions and talc insufflations through thoracoscopy is currently the best method for chemical pleurodesis [10]. However, there are serious concerns about its safety. Most significantly is adult respiratory distress syndrome which occurs in as many as 9% of patients receiving intra-pleural talc [11]. Systemic embolization of talc has been shown in animal studies and in humans. The non-availability and cost of medical talc remains a constraint for poor patient in developing countries [12]. Tetracycline is an effective agent for pleurodesis with an average success rate of about 77% [10,13,14]. The advantages of tetracycline are reasonable efficacy, excellent safety profile, ease of administration and low cost. Adverse effects of tetracycline include pain and fever. The main encumbrance with its use is that the production of its parenteral form has been discontinued, although tetracycline powder (suspension) obtained from tetracycline capsules are commonly used [13]. It is the commonest agent used in most centres in Nigeria [15].

Povidone-iodine is a topical antiseptic that has been reported as a promising agent for pleurodesis [16]. It is readily available and cheap. Some reports have recorded success rates of 98.4% using 10% povidone [8,17]. Different concentrations have also been used 2%, 4% with similar success rates and safety profile [18]. The most common adverse effect is pleuritic chest pain. In a meta-analysis by Agwaral *et al*. hypotension was reported in 6 patients amongst 499 patients in all the studies analysed. This was associated with chest pain and possibly due to vaso-vagal reflex [9]. However, reports of rare occurrence of thyroiditis and visual loss have been reported after instillation of large volumes (200-500ml) of 10% povidone-iodine, this has not been noted in subsequent studies using lower concentrations (5%, 4% or 2%) and smaller volumes of povidone iodine [9,19]. Although, there has been no report of povidone iodine use for pleurodesis in Nigeria. Bleomycin is an antibiotic agent that is commonly used in some countries (example Iran) for pleurodesis [20]. However, it is expensive compared to other agents thus limiting its use. Dextrose 50%, transforming growth factor beta (TGF-b), doxycycline, olive oil, minocycline, corynebacterium parvum extract, interferons (α and β), interleukins (IL-2) and several chemotherapeutic agents such as cisplastin, cytosine arabinoside and mitoxantrone have been used, though with variable efficacy rates [10,21-23]. In Nigeria the burden of malignant pleural effusion is huge and similar to what obtains in other developing countries.

This is due to the fact that majority of cancer cases presents with advanced stages of the disease [6,7]. Further, with insufficient number of thoracic surgeons in Nigeria and most developing countries, most patients are managed my respiratory physicians and medical officers. This causes delay in diagnosis due to the fact that symptoms of lung cancer and pulmonary tuberculosis (which has a high prevalence) are similar, and most patients receive anti-tubercular therapy for varying periods before their definitive diagnosis. Diagnostic facilities like fiberoptic bronchoscopy, fine needle aspiration cytology/biopsy are not uniformly available throughout the country and even in centers where they are available, lack of experienced pathologist in reporting on cytology specimens may be responsible for diagnostic delays [24]. With most patients presenting in advanced stages of malignancy (malignant pleural effusion being the predominant presenting complication), and associated co-mobidities such as cardiovascular diseases most patients are not fit for radical surgical therapies. Thus, the main treatment is palliative which would include pleurodesis. The commonest agent used in Nigeria is tetracycline capsules (constituted into a suspension). Povidone iodine has been used for pleurodesis in some European, Middle Eastern and Asian countries with superior success rates (when compared to tetracycline) and safety profile. However, there are little or no studies of its use in Nigeria. Its relative availability, low-cost and definite concentrations makes it an attractive alternative to tetracycline. Sterile talc costs about $111.99 (USD) for a 5g ampoule, whilst povidone iodine costs about 580 Naira (equivalent to $1.5 USD) for a 100ml solution. The aim of this study is to assess the efficacy and safety of povidone iodine in producing pleurodesis. The specific aims are listed below: i) to determine the efficacy of 5% povidone-iodine compared with tetracycline suspension (control group) in producing pleurodesis in patients with malignant pleural effusion; ii) to identify adverse effects of these agents when used for pleurodesis.

## Methods

**Study population and design:** this study was carried out in the cardiothoracic surgery unit of the university of Benin Teaching Hospital, Benin-city, Edo State, Nigeria [25]. The study included all patients admitted with symptomatic malignant pleural effusion over a period of 1 year. This included referrals from other units within the hospital. Over a period of 12 months, 30 patients were recruited into the study. The study is a prospective experimental study. The patients are randomized into two groups A (control) and B. Group A patients (control) received tetracycline treatment while group B received povidone iodine treatment. Patients are assigned into each group consecutively as they presented starting from group A. Informed consent was obtained from each patient.

**Inclusion criteria:** i) all patients with symptomatic malignant pleural effusion established by positive result of pleural effusion cytology and/or pleural biopsy; ii) chest radiograph confirming full lung expansion.

**Exclusion criteria:** i) patients with bleeding disorders; ii) trapped lung; iii) extensive thoracic skin infiltration by tumour; iv) known hypersensitivity to iodine; v) patients with thyroid disease; vi) patients with very limited life expectancy.

**Materials:** these included a chest tube, drainage bottle, local anaesthetic (xylocaine), suturing pack (consisting of needle holder, dissecting forceps, clamps, gallipots, sponge forceps), 50ml syringe, sutures of various sizes (non absorbable), povidone iodine 5%, tetracycline capsules, normal saline infusion. Demographic and anthropometric data of patients were collected including age, sex, duration of pleural effusion, presenting symptoms, number of previous thoracocentesis procedures, and type of primary malignancies.

**Ethical consideration:** ethical approval was sought for this study from the Ethics and Research Committee of the University of Benin Teaching Hospital, Benin-city, Edo State.

**Pleurodesis procedure:** a chest tube (28fr) is inserted through the 5^th^ intercostal space mid axillary line and connected to an under-water sealed tube drainage to achieve complete drainage of the effusion and lung re-expansion. The drainage should be controlled to avoid the development of re-expansion pulmonary oedema. The patient commences chest physiotherapy with an incentive spirometer to encourage lung re-expansion. As soon as the drainage is less than 100mls/24hours and the lung fully re-expanded (confirmed with radiograph), pleurodesis is performed at the patient´s bedside. Twenty (20) ml of 2% lidocaine is diluted with 30ml of normal saline. Tetracycline powder from tetracycline capsules 1g is mixed with the pre-mixed 50ml solution of lidocaine and saline and instilled into the pleural cavity through the chest tube using a 50ml syringe in group A patients. Group B patients received a mixture of 20ml 5% povidone iodine mixed with the pre-mixed 50ml solution of Lidocaine and saline instilled through the chest tube into the pleural cavity. The chest tube is clamped for 2hours and patient is advised to rotate every 30minutes into the left lateral, right lateral, prone and supine positions thereafter the tube is released. If the post procedure drainage is less than 100mls per day, the tube is removed and the patient observed as an out-patient.

**Evaluation and monitoring:** any complication related to the procedure were recorded and managed. All patients are assessed with chest X-ray after one (1) week and one (1) month. The chest X-rays were read by a consultant radiologist who was “blinded” to the different patients groups. The same radiologist read the chest X-rays of patients from both groups. The response of this procedure, treatment failure and complaints of the patients were evaluated (Table 1).

**Table 1:** response to treatment

Complete response	Symptomatic improvement of dsypnoea with complete radiographic resolution of the pleural effusion within 30 days.
Partial response	Symptomatic improvement with recurrent pleural effusion that did not require additional thoracocentesis.
Treatment failure	Recurrent pleural effusion that required thoracocentesis.

**Data analysis:** the data obtained were analysed using the statistical package for social studies (SPSS) version 22. A value of p<0.05 is considered as statistically significant.

## Results

**Figure 1:**
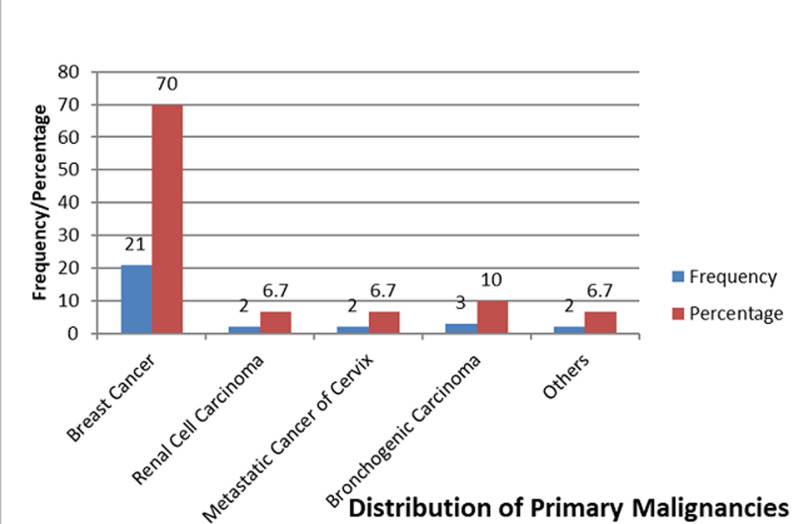
primary malignancies

Thirty (30) patients were recruited into this study, 15 patients in each group A (tetracycline) and B (povidone iodine). The mean age was 45.7±14.24 years. Twenty five (83%) of these patients were women while five (17%) were men. The distribution of the occupations of the patients showed that 17(57%) were into business, 6(20%) were civil servants, 7(23%) comprised of a student, one retired patient whilst the others were unemployed. The commonest primary malignancy was breast cancer (70%) followed by bronchogenic cancer (10%) (Figure 1). Dyspnoea was the most common symptom amongst the patients occurring in 96.7% of the patients, other symptoms included chest pain (90%), cough (83.3%) and hemoptysis (26.7%) (Table 2). Thoracocentesis yielded serous fluid in 60% of the patients while others had hemorrhagic fluid 33.3% and turbid fluid 6.7%. Pleural aspirate cytology was positive in 66.7% of patients whilst pleural biopsy histology was positive in 43.3% of patients. Following pleurodesis, only six patients (20%) had complaints of chest pain whilst others had no complaints. The mean volume of fluid drained daily was 75.6±36.26, however, the mean volume drained in the povidone group was 70.7±24.6 and that of the tetracycline group was 80.7±45.4 (p-value=0.129). Most patients were extubated on the second day post pleurodesis. However, the mean days before extubation in the povidone iodine group was 2.8667±2.8752 and the tetracycline group was 2.1333±0.9904 (p=0.236). Seventy-three-point three percent (73.3%) (22) of these patients in this study had complete response after pleurodesis as shown in Table 3. P-value: 0.716 (levene´s test for equality of variance), statistically using the levene´s test for equality of varience, there was no significant difference between volume of pleural fluid drained post pleurodesis and the agent used (p=0.732). It was also noted that gender, nature of pleural fluid drained, post procedure complaints or primary malignancy did not vary with any statistical significance to the response based on the different agents.

**Table 2:** presenting symptoms of patients

Symptoms	Number of patients	Percentage
**Dyspnoea**	29	97%
**Cough**	27	90%
**Chest pain**	25	83%
**Hemoptysis**	8	27%

**Table 3:** response versus agent used

		Agent used		Total
Response		Povidone	Tetracycline	
	Complete	10 (66.7%)	11 (73.3%)	21
	Partial	4 (26.7%)	3 (20%)	7
	Failure	1 (6.7%)	1 (6.7%)	2
Total		15	15	30

## Discussion

Malignant pleural effusion (MPE) occurs as a consequence of a primary malignancy of the pleura or secondary involvement of the pleura from a metastatic malignant process. Management of MPE is mostly palliative as MPE represents an advanced stage of malignancy. Palliative measures include pleurodesis which involves the use of several methods most commonly the use of sclerodesing agents. These agents include talc, tetracycline, doxycycline, bleomycin. In Nigeria, tetracycline (capsules) is the commonest method used, medical grade talc is expensive and in most cases not available for use in most centers. Povidone iodine is a common agent in most Nigerian hospitals and it is used routinely for wound dressings, as a scrubbing agent and for patient´s skin preparation in theatre. There has not been any documented evidence of its use in Nigeria as a pleurodesing agent, although its use has been routine for over a decade in South America and some Asian countries. In this study, 30 patients were recruited and randomized into two groups A (control-tetracycline) and B (povidone iodine). Five percent (5%) povidone iodine (WOSAN) was used as described in the methodology. The mean age was 45.7±14.24 years and the youngest patient was a 10 year old boy with a primary malignancy of osteosarcoma. This shows that MPE affects a wide age range and thus management strategies must be instituted to palliate these patients. Eighty-three percent of the patients were women; this is not surprising because the most common primary malignancy was breast cancer (70%) which is still the leading cancer in Nigeria especially amongst women [26,27], this was at par with findings by Tettey *et al*. in Ghana which women made up the all the patients who had pleurodesis over the four year study and breast cancer was the commonest malignancy [13].

Most patients were businessmen/women (17 patients), representing an economic viable population in the community and also are the providers for their families thus would be devastating for their families and the economy to lose them to recurring hospital admissions due to MPE or eventually death. This essentially supports the aim of this study to find another affordable, widely available and effective pleurodesing agent as a supplement/alternative to tetracycline. Bronchogenic cancer was just about 10% of the primary malignancies in this study which is in variance with studies by Olivares *et al*. in Mexico and other studies from Iran, Japan and India [8,28,29] where bronchogenic cancer made up more than 30% of primary malignancies causing MPE. Although a study from Ghana over a 4-year period did not have a single patient with MPE who had a primary malignancy of bronchogenic cancer [13]. This is not surprising because though the incidence of bronchogenic cancer is increasing stemming from an increase number of women and young people who smoke in Nigeria and worldwide, breast cancer is still the most prevalent cancer in Nigeria especially amongst women and in sub-Saharan Africa [30]. Dyspnoea was the most common symptom present in almost all patients (97%) closely followed by cough (90%) and chest pain (83%), this is similar to findings by Tettey *et al*. in Ghana where dyspnoea was present in all patients in their study although, chest pain was the next most common symptom [13].

Only 20% of these patients had complaints of pain and this was evenly distributed between both groups of patients. This is similar to studies using similar agents as noted in a systematic review by Shaw and Agwaral, Kahrom *et al*. and also in a study by Olivares *et al*. [2,8,31]. These patients had analgesia with xylocaine during the procedure, the post procedure pain was mild and required only mild analgesia (paracetamol). It was noted that the post procedure pleural fluid drainage of the povidone iodine group was lower than that of the tetracycline group by about 10mls although this wasn´t statistically significant. Indeed a significant number of the patients were extubated on the second day post procedure, those who had tetracycline were found to be extubated a bit earlier when compared to the povidone iodine group. Seventy-three percent (73%) of the patients in this study had complete response and in 7% pleurodesis failed whilst 20% has partial response. Thus, overall success rate is 93%. In addition, in the povidone group the overall success rate was 93.4% and in the tetracycline group it was 93.3% with a p-value of 0.716. There was no statistical difference in the responses based on the agents used. This shows the efficacy of povidone iodine as an alternative to tetracycline for use as a pleurodesing agent. This is validated by similar findings which showed remarkable success rates to povidone iodine such as Olivares *et al*. (96.1%), Godazandeh *et al*. (91.6%), Dey *et al*. (89.5%), and Shaw and Agwaral (88.7%) [2,8,28,29]. There also were no statistical differences between gender, nature of fluid drained, post procedure complaints and volume of fluid drained to the response of both agents.

## Conclusion

Malignant pleural effusion is a devastating condition as it heralds the end of life processes of a primary malignancy. It takes a huge toil on the patients and also the hospital due to recurrent admission for pleural fluid drainage, these days spent on admission consumes vital time from these patients that could have be put into economic profiting ventures and it also puts pressure on the lean hospital beds and also the surgeon. Pleurodesis is an effective palliative treatment that prevents reaccumulation of pleural fluid thus preventing recurrent hospital admissions. Talc is the commonest agent used in most western countries, however, tetracycline (capsules) is the most common agent used in Nigeria and in West Africa. Povidone iodine 5% is an agent which has been in use for wound dressings, hand scrubbing and for skin prep for surgery. Its use as a pleurodesing agent has been documented to be remarkably effective as seen in studies in Asia and South America. This study has shown that it is equally as effective as tetracycline in producing effective pleurodesis in patients with MPE. Finally, povidone iodine is a safe, effective, cheap, widely available and effective pleurodesing agent for use in patients with malignant pleural effusion.

### What is known about this topic

- Talc is the most effective pleurodesing agent used, however, its cost makes it not assessible to poor sub-Saharan countries;
- Povidone iodine has been used in the middle east and Asia with good outcomes but no study (to my knowledge) about its use in Nigeria.

### What this study adds

- It shows that povidone iodine is as effective for pleurodesis when compared to tetracycline which is the mostly used agent in Nigeria;
- This adds to pool of information regarding effective pleurodesing agents that can be used in patients with malignant pleural effusion as there are few publications on this topic from Nigeria.
